# Supplementary material for: Proteomics- and Metabolomics-Based Analysis of Metabolic Changes in a Swine Model of Pulmonary Hypertension
Source: Int J Mol Sci. 2023 Mar 2;24(5):4870. doi: 10.3390/ijms24054870 (PMC10003314; doi:10.3390/ijms24054870)
Supplement: Supplementary file 1 [file ijms-24-04870-s001.zip › Supplementary figures.pptx]

## Slide 1
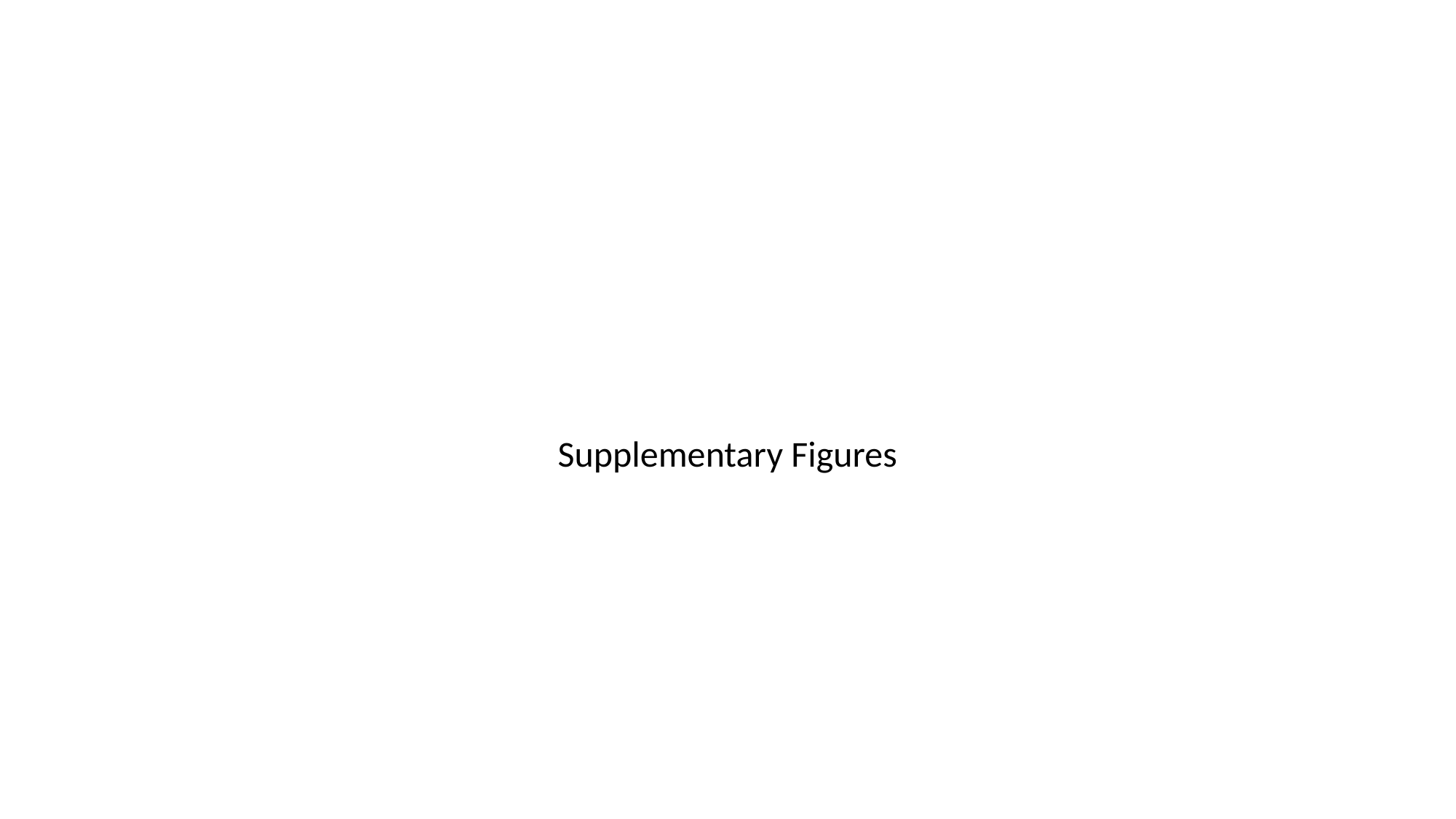

Supplementary Figures

## Slide 2
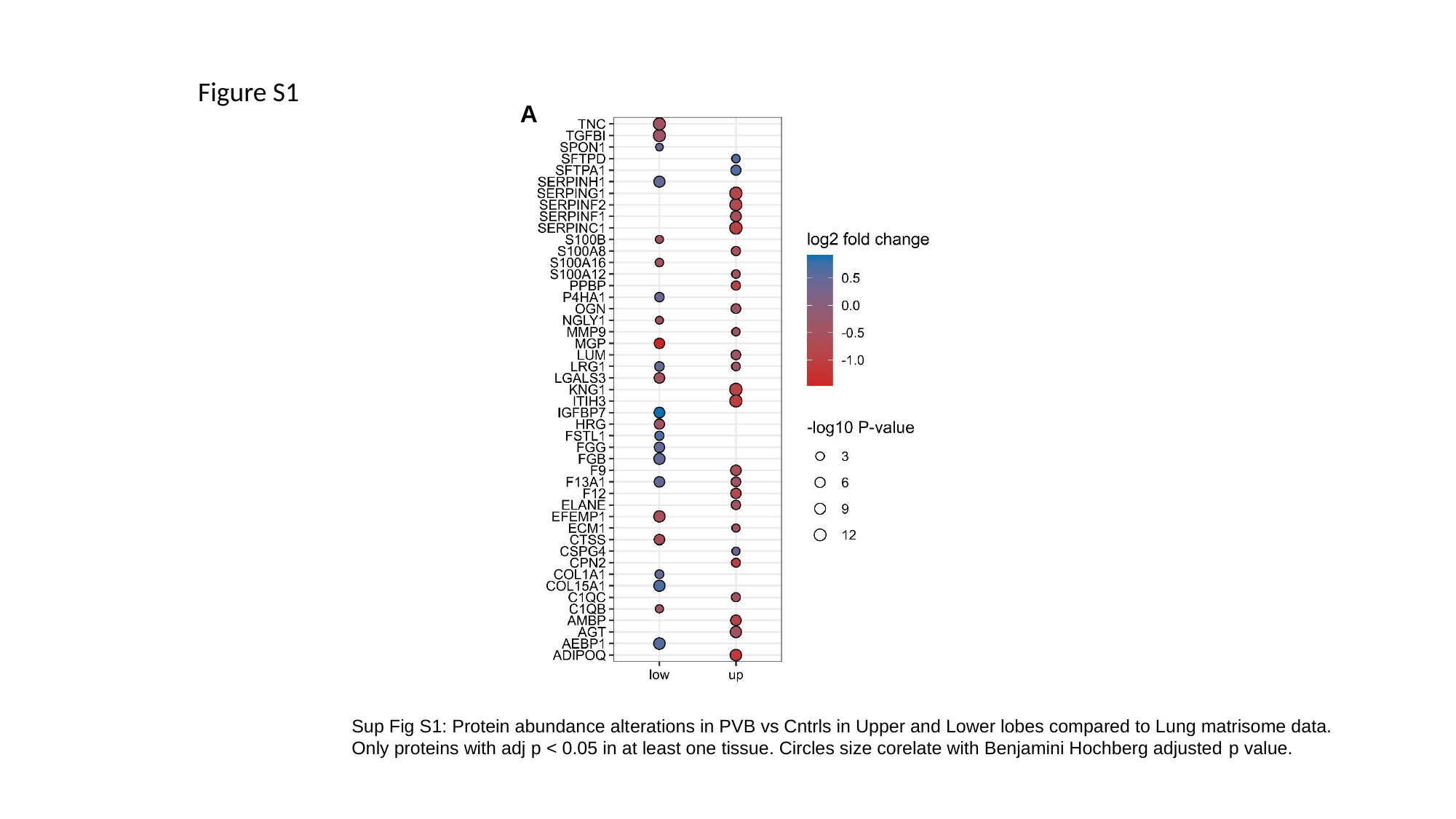

Figure S1
A
Sup Fig S1: Protein abundance alterations in PVB vs Cntrls in Upper and Lower lobes compared to Lung matrisome data.
Only proteins with adj p < 0.05 in at least one tissue. Circles size corelate with Benjamini Hochberg adjusted p value.

## Slide 3
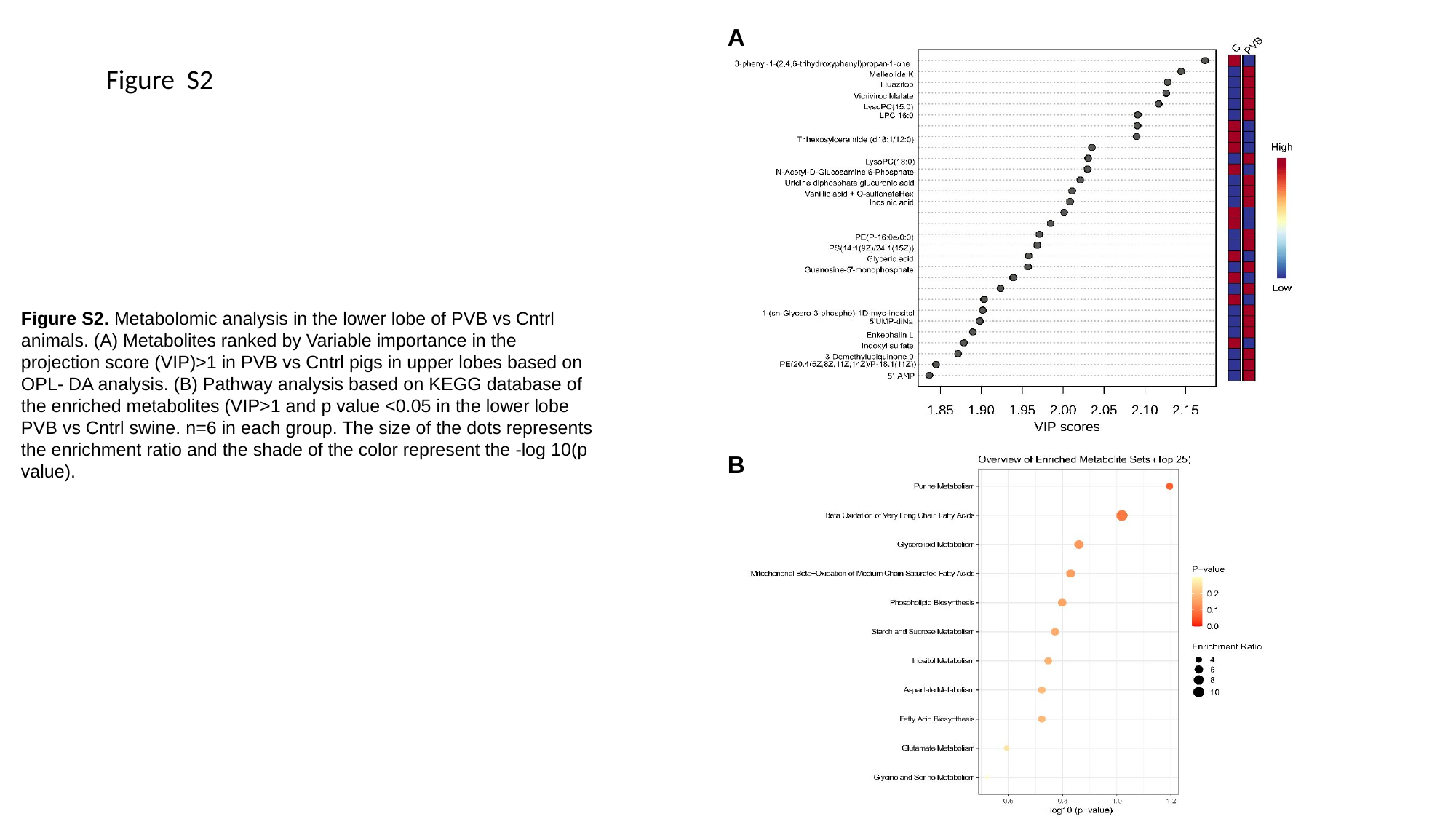

A
Figure S2
Figure S2. Metabolomic analysis in the lower lobe of PVB vs Cntrl animals. (A) Metabolites ranked by Variable importance in the projection score (VIP)>1 in PVB vs Cntrl pigs in upper lobes based on OPL- DA analysis. (B) Pathway analysis based on KEGG database of the enriched metabolites (VIP>1 and p value <0.05 in the lower lobe PVB vs Cntrl swine. n=6 in each group. The size of the dots represents the enrichment ratio and the shade of the color represent the -log 10(p value).
B

## Slide 4
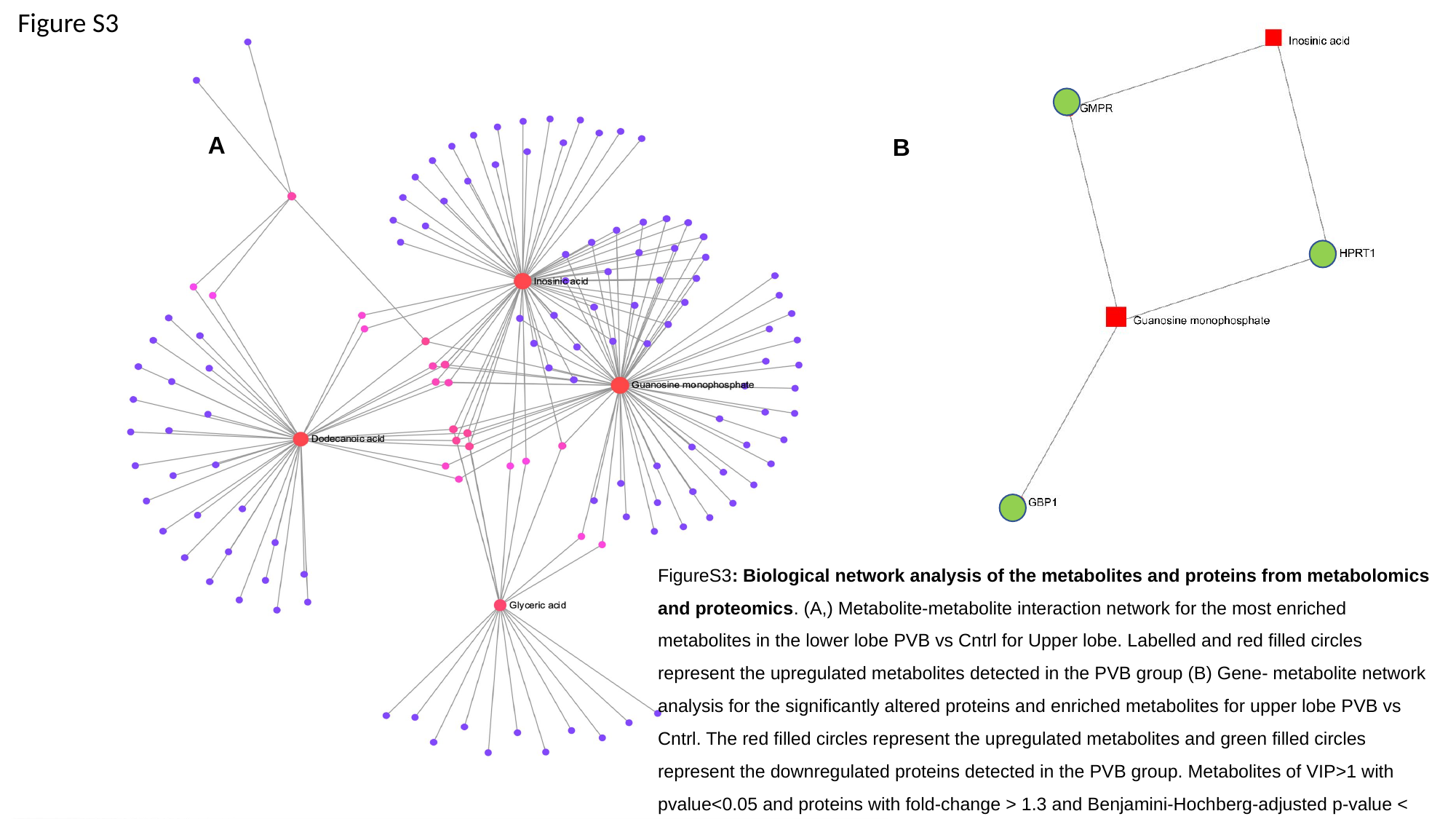

Figure S3
A
B
FigureS3: Biological network analysis of the metabolites and proteins from metabolomics and proteomics. (A,) Metabolite-metabolite interaction network for the most enriched metabolites in the lower lobe PVB vs Cntrl for Upper lobe. Labelled and red filled circles represent the upregulated metabolites detected in the PVB group (B) Gene- metabolite network analysis for the significantly altered proteins and enriched metabolites for upper lobe PVB vs Cntrl. The red filled circles represent the upregulated metabolites and green filled circles represent the downregulated proteins detected in the PVB group. Metabolites of VIP>1 with pvalue<0.05 and proteins with fold-change > 1.3 and Benjamini-Hochberg-adjusted p-value < 0.05 were used for the analysis.

## Slide 5
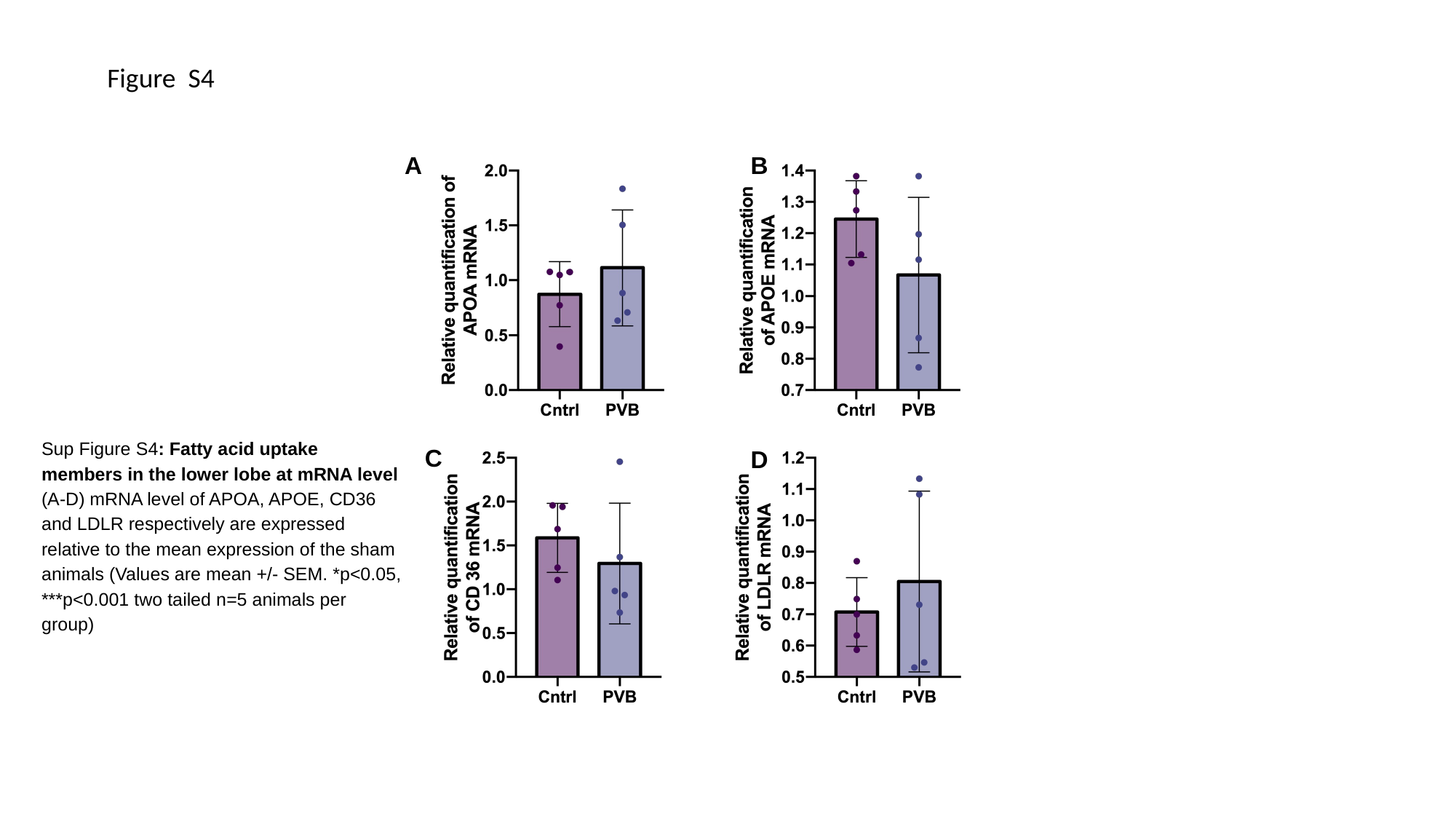

Figure S4
B
A
Sup Figure S4: Fatty acid uptake members in the lower lobe at mRNA level (A-D) mRNA level of APOA, APOE, CD36 and LDLR respectively are expressed relative to the mean expression of the sham animals (Values are mean +/- SEM. *p<0.05, ***p<0.001 two tailed n=5 animals per group)
C
D
